# Achieving Ultra-Low Friction with Diamond/Metal Systems in Extreme Environments

**DOI:** 10.3390/ma14143791

**Published:** 2021-07-07

**Authors:** Pantcho Stoyanov, Rolf Merz, Markus Stricker, Michael Kopnarski, Martin Dienwiebel

**Affiliations:** 1 MicroTribology Center µTC, Fraunhofer-Institute for Mechanics of Materials IWM, Wöhlerstrasse 11, 79108 Freiburg, Germany; martin.dienwiebel@kit.edu; 2 Institute for Applied Materials IAM, Karlsruhe Institute of Technology KIT, Kaiserstrasse 12, 76131 Karlsruhe, Germany; markus.stricker@rub.de; 3 Department of Chemical and Materials Engineering, Concordia University, 1455 De Maisonneuve Blvd. W., EV 2.285, Montreal, QC H3G 1M8, Canada; 4 IFOS, “Institut für Oberflächen- und Schichtanalytik GmbH”, Trippstadter Straße 120, 67663 Kaiserslautern, Germany; merz@ifos.uni-kl.de (R.M.); kopnarski@ifos.uni-kl.de (M.K.); 5 Interdisciplinary Centre for Advanced Materials Simulation, Ruhr-University Bochum, Universistätsstraße 150, 44801 Bochum, Germany

**Keywords:** space tribology, interfacial phenomena, diamond, tungsten, third-body, XPS, AES, scaling effects

## Abstract

In the search for achieving ultra-low friction for applications in extreme environments, we evaluate the interfacial processes of diamond/tungsten sliding contacts using an on-line macro-tribometer and a micro-tribometer in an ultra-high vacuum. The coefficient of friction for the tests with the on-line tribometer remained considerably low for unlubricated sliding of tungsten, which correlated well with the relatively low wear rates and low roughness on the wear track throughout the sliding. Ex situ analysis was performed by means of XPS and SEM-FIB in order to better understand the underlying mechanisms of low friction and low-wear sliding. The analysis did not reveal any evidence of tribofilm or transferfilm formation on the counterface, indicating the absence of significant bonding between the diamond and tungsten surfaces, which correlated well with the low-friction values. The minimal adhesive interaction and material transfer can possibly be explained by the low initial roughness values as well as high cohesive bonding energies of the two materials. The appearance of the wear track as well as the relatively higher roughness perpendicular to the sliding indicated that abrasion was the main wear mechanism. In order to elucidate the low friction of this tribocouple, we performed micro-tribological experiments in ultra-high vacuum conditions. The results show that the friction coefficient was reduced significantly in UHV. In addition, subsequently to baking the chamber, the coefficient of friction approached ultra-low values. Based on the results obtained in this study, the diamond/tungsten tribocouple seems promising for tribological interfaces in spacecraft systems, which can improve the durability of the components.

## 1. Introduction

The endurance life of various components within spacecraft systems is limited by the tribological performance of the employed materials due to the large number of complex contacting and moving assemblies [1]. Solid lubricants are commonly used in such interfaces with the purpose of helping to overcome many of the aerospace challenges ranging from improving durability of the spacecraft to developing novel mechanical systems [1]. However, degradation of solid lubricants due to the large fluctuations in demanding conditions can lead to premature failure of the component and consequently severe damage to the assembly. Therefore, there is a strong desire to develop alternative tribocouples that can operate effectively over billions of cycles in harsh environments, while also being capable of surviving the fluctuating conditions during operation. 

Carbon-based materials have received a lot of attention due to their high potential for reducing friction and wear in aerospace applications [2,3,4,5,6,7,8]. Evidently, the high hardness, wear resistance, and chemical inertness of diamond makes it extremely attractive for such applications, ranging from mechanical seals [4] to nano-/micro-electromechanical systems [9]. Indeed, nano- and microstructured bulk diamond and diamond coatings are currently being used in such applications, and are commonly grown using various chemical vapor deposition (CVD) methods (e.g., plasma-enhanced CVD, hot-filament CVD and laser-assisted CVD). 

Similar to other sliding systems, the tribological response of such carbon-based materials has been shown to be governed by the interfacial process, which generally consists of phase transformations, mechanical mixed layers and/or transfer films, as well as the formation of the debris particles [2,3,10,11,12,13,14,15,16]. Thus, several research efforts have been made in order to understand the structure and properties of these so-called third bodies and to correlate them to the friction and wear behavior [5,17,18,19,20,21]. Due to the confined nature of the sliding contact, it is difficult to access the sliding interface and, consequently, most of our understanding is based on ex situ techniques. Even though valuable knowledge has been obtained using these techniques it is certainly possible that different dynamic events at the sliding interface, causing fluctuations in the friction and wear, remain undiscovered. 

In a relatively recent review article, Sawyer et al. [22] summarized some of the most common techniques used to monitor third-body dynamic events during sliding. These techniques are useful in particular for understanding the interfacial processes of carbon-based materials. For instance, in situ tribometry (i.e., within the contact) has previously been used to monitor the velocity accommodation modes (VAMs) at the sliding interface of nanocrystalline diamond coatings [23]. Using this macro-scale in situ examination with a sapphire counterface, the authors found that the main VAM was interfacial sliding of a carbonaceous transfer film versus the wear track of the coating. At the atomistic scale, however, novel experimental techniques and computational methods have made it possible to obtain important atomistic insights into the sliding of carbon-based materials [17,24,25,26,27,28,29]. Gao et al. [25] performed atomic force microscopy (AFM) and molecular dynamics (MD) simulations to examine the friction and surface orientation of diamond. The authors found that the nanoscale tribological behavior deviates from the macro-scale sliding behavior of diamond. 

In a series of previous studies on different carbon-based materials [30,31,32], we used a novel approach by linking the techniques mentioned above (i.e., ‘on-line’ macroscale sliding experiments and ex situ analysis with atomistic simulations) in order to contribute to a better understanding of the third-body evolution in tribological interfaces. The aim of this paper particularly is to use the methodologies mentioned above to investigate the tribological behavior of diamond in contact with tungsten and to understand if ultra-low friction can be achieved under un-lubricated sliding conditions. The experiments were performed using the on-line tribometer (i.e., within the environment), while the ex situ analysis of the counterbodies was performed by means of X-ray photoelectron spectroscopy (XPS) in order to capture the chemical interaction of the interfacial processes. Then, we performed experiments in an ultrahigh vacuum in order to understand the performance of this tribocouple under spacecraft relevant conditions. 

## 2. Methods

**Figure 1:**
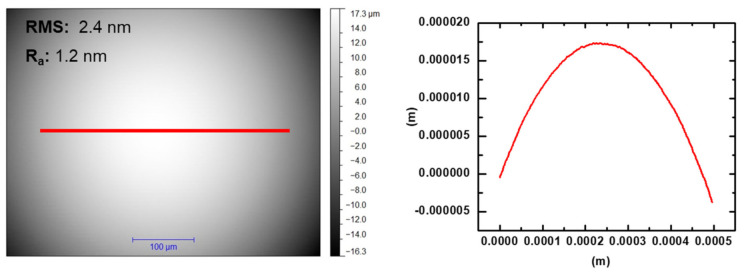
Diamond tip used for the on-line tribological testing in this study. The roughness (Ra) of the diamond tip was measured to be 1.2 nm by means of atomic force microscopy (AFM).

In this study, macroscale tribology experiments were performed using an on-line tribometer, equipped with a force sensor, a holographic microscope and an atomic force microscope, allowing for monitoring of topographical changes after each cycle. The instrument is described in detail elsewhere [33]. The experiments were performed on a 99.9 wt.% tungsten plate obtained from Goodfellow GmbH in the ‘as rolled’ condition and polished down to an RMS roughness of 34 ± 4 nm. Cross-sectional SEM images obtained from focused ion beam (FIB) cuts of the sample prior to the sliding experiments revealed that the polishing procedure did not have a significant influence on the near-surface structure [34]. Similarly, X-ray photoelectron spectroscopy (XPS) analysis of the unworn surface showed no significant changes in the chemical composition (e.g., oxide layers) after the polishing procedure [34]. A diamond sphere obtained from Synton-MDP LTD (Nidau, Switzerland) with a radius of 1.5 mm was used as the counterface for the sliding tests. The roughness (Ra) of the diamond tip was measured to be 1.2 nm by means of atomic force microscopy (AFM), as shown in Figure 1. The experiments were performed with a sliding velocity of 5 mm/s and an initial normal load of 2 N in reciprocating mode. These parameters were chosen based on the capability of the on-line tribometer. Microtribology experiments in an ultrahigh vacuum (pressure of 10^−7^ Pa) were also performed and compared to sliding tests performed at 50% relative humidity. The average friction value presented in the figure is based on three tests for each condition. Additional microtribology experiments were also performed after baking the chamber for more than 24 h. Ex situ chemical and structural analysis of the worn surfaces (i.e., plates and counterfaces) was performed using XPS (Axis Nova, Kratos Analytical Inc., Manchester, UK), Auger electron spectroscopy (Smart 200, Physical Electronics, Inc., Chanhassen, MI, USA). Lateral elemental concentration distributions were also obtained by parallel imaging XPS spectroscopy. The XPS results were compared to analysis performed on the unworn surface using the same methodology. 

## 3. Results and Discussion

### 3.1. Macroscopic Sliding Experiments

**Figure 2:**
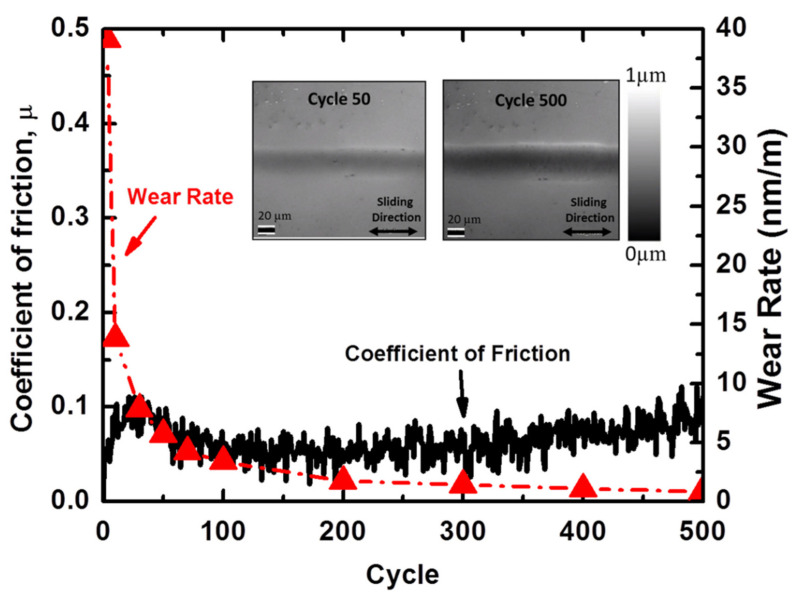
Coefficient of friction and wear rate vs. cycle for diamond sliding against tungsten under unlubricated conditions using a macro-scale tribometer.

The results from the on-line sliding tests of diamond against tungsten in air are presented in Figure 2. The friction coefficient in the dry sliding experiments revealed a certain running-in phase; initially the coefficient of friction increased up to 0.1 after approximately 25 cycles followed by a decrease to approximately 0.07 and subsequently an increase up to a value of 0.12. It should be noted that the coefficient of friction observed in these experiments is considerably low for unlubricated sliding of tungsten against diamond.

**Figure 3:**
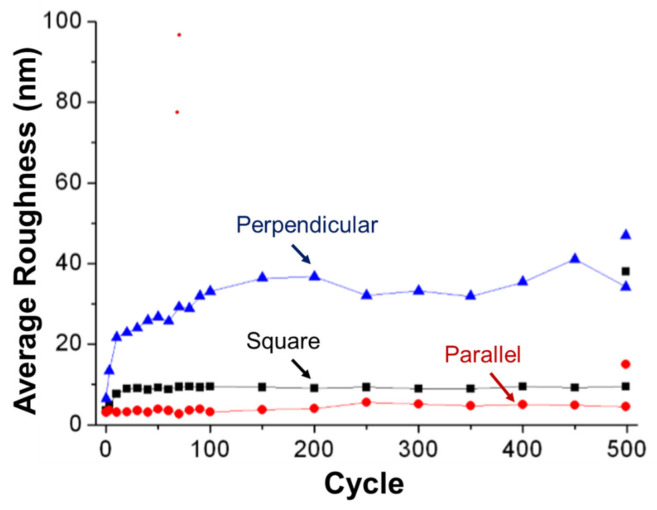
Average roughness for diamond sliding against tungsten using a macro-scale tribometer.

One benefit of using an on-line tribometer is the capability to measure the wear rates and roughness (i.e., in the parallel and perpendicular directions of sliding) at any given cycle, using the holographic images as shown in Figure 3. The average roughness evolution in the parallel direction throughout sliding remained constant and below 10 nm throughout the whole sliding process while the roughness in the perpendicular direction increased upon initial sliding up to a value of ~40 nm, after which it remained nearly constant for the remainder of the experiment (see Figure 3). This difference in average roughness between the parallel and perpendicular directions has previously been observed in metallic sliding contacts and is attributed to the adaptation process of the two surfaces [31]. 

The wear rates throughout the sliding process are also shown in Figure 2. The wear rate was initially ~40 nm/m and then decreased to below 5 nm/m within the first 70 cycles. Subsequently, the wear rate continued to decrease to rates below 1 nm/m, indicating ultra-low wear. The wear behavior correlates well with the variation in roughness throughout sliding. More specifically, the increase in roughness perpendicular to the sliding within the first few cycles indicates that the main wear mechanism is abrasion and most of the wear occurs at the beginning of the test. 

### 3.2. Microscopic Sliding Experiments

**Figure 4:**
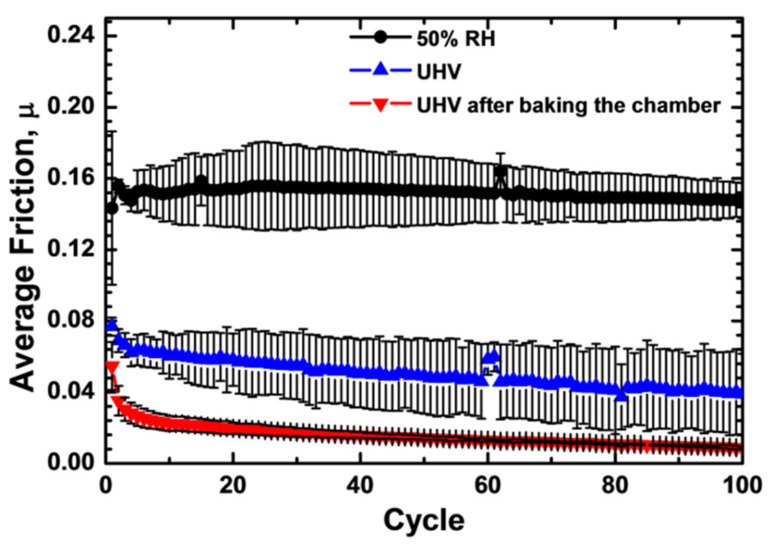
Average friction coefficient vs. cycle for diamond/tungsten sliding tests using a microtribometer. The experiments were performed at 50% relative humidity and compared to tests in UHV as well as after baking in the chamber for more than 24 h.

Microtribology tests were also performed in UHV and compared to the dry sliding tests at 50% relative humidity as shown in Figure 4. Clearly, the average friction was significantly reduced in the ultrahigh vacuum compared to the 50% RH experiments. In addition, micro-scale sliding experiments performed in UHV after baking the chamber for more than 24 h further reduced the friction coefficient. Evidently, this behavior is associated with the reduction in the water condensation on the surfaces, which minimizes the capillary forces and thus, the resistance to movement. Such influence of humidity on the coefficient of friction due to capillarity forces is common in microscale sliding contacts and has previously been reported [35,36,37]. It should be noted that there is also an adequate amount of tribochemistry occurring between water and carbon materials, which can contribute to an increase in friction. 

### 3.3. Chemical and Structural Analysis 

**Figure 5:**
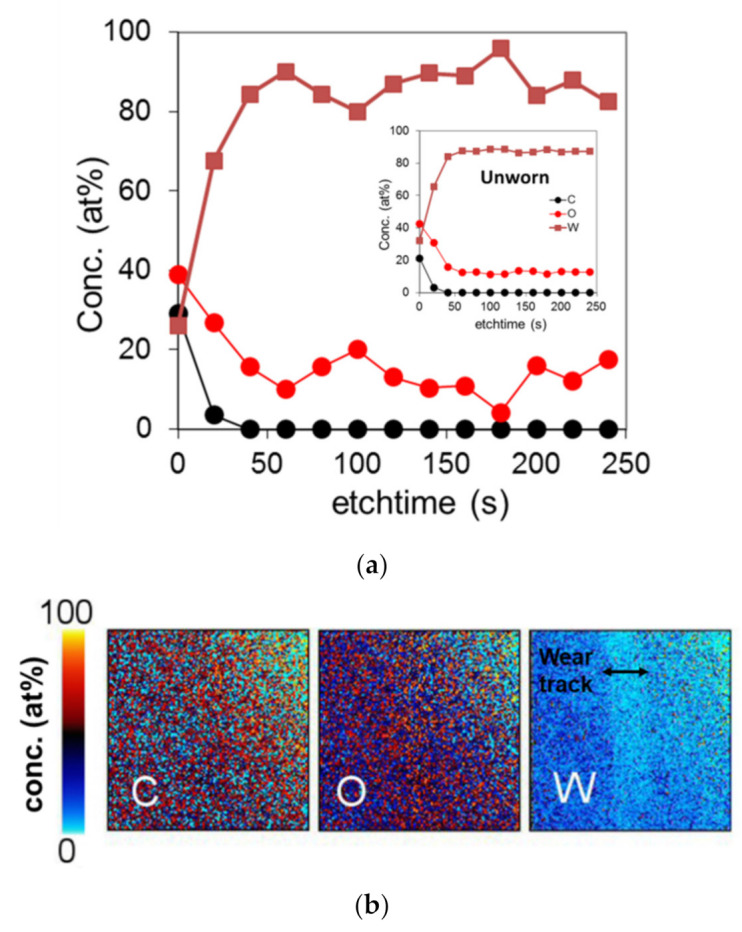
(**a**) XPS depth profile of the worn tungsten surface under dry sliding conditions, including the XPS depth profile of the unworn tungsten surface as a reference. (**b**) Elemental map of the wear track.

In order to provide a better understanding of the interfacial processes leading to ultra-low friction and wear behavior in the macroscale sliding system, we performed a detailed structural and chemical ex situ analysis by means of XPS. Figure 5 shows the chemical analysis for the worn and unworn tungsten surface. The XPS analysis in Figure 5a as well as the elemental map in Figure 5b show no significant increase in the oxide content for the worn surface compared to the unworn surface (see inset in Figure 5a for the depth profile in the unworn region). It should be noted that this is not typical for metallic sliding contacts, where an increased oxygen content near the surface has been reported numerous times [34,38,39,40]. Interestingly, the XPS analysis shows no significant difference in the carbon concentration (i.e., for the tungsten, carbon, and oxygen) between the worn and the unworn surface, indicating no evidence of the formation of a carbon-based layer. 

**Figure 6:**
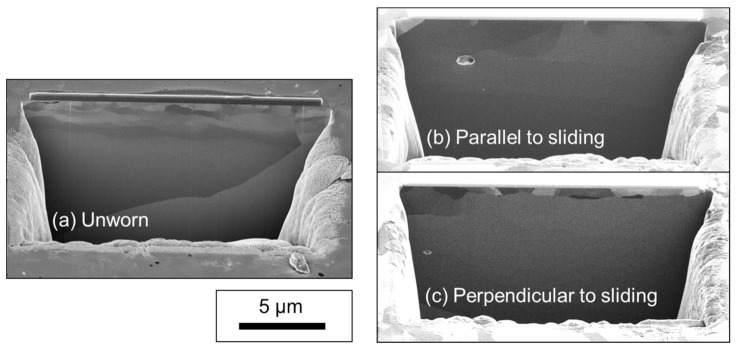
SEM/FIB cross-sectional image of (**a**) the unworn tungsten surface and (**b**) the worn tungsten surface in the parallel and (**c**) in the perpendicular direction generated using an on-line macro-tribometer.

Figure 6 shows the structural analysis by means of cross-sectional SEM images of the worn tungsten surface created using the on-line tribometer. A 1-µm-thick grain refined layer is clearly visible in the subsurface region; however, it is somewhat similar in appearance to the one for the unworn surface. Some degree of subsurface grain refinement is typical for microcrystalline metal sliding contacts and can be attributed to nucleation of dislocations and possibly rotation of grains [40,41]. 

**Figure 7:**
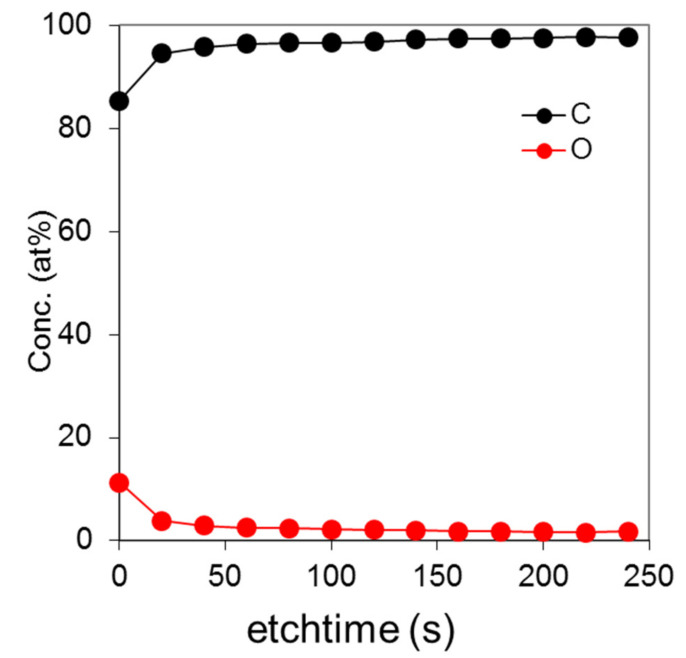
XPS depth profile of the diamond tip subsequent to sliding against tungsten.

Ex situ analysis of the diamond counterface after sliding was performed in a similar way as for the tungsten specimen. The XPS depth profile in Figure 7 shows predominately carbon with some small traces of oxygen. In addition, the XPS depth profile of the worn diamond tip showed no evidence of tungsten; however, there was a slight increase in the oxygen content on the surface. 

The ex situ analysis performed in this study revealed no significant evidence of the formation of a tribofilm or a transferfilm on the diamond counterface. This indicates the absence of significant bonding between the diamond and tungsten surface, which correlated well with the low friction values. It should be noted that such minimal adhesive interaction and low friction are not typical for metal/ceramic type of interfaces under these sliding conditions [31,32,34]. The minimal adhesive interaction and material transfer with this tribocouple can possibly be explained by the high cohesive bonding energies of the two materials, as well as the low initial roughness of the two surfaces, which has previously been shown to be a critical factor in reducing friction for diamond/metal interfaces [42]. However, when running ground diamond against steel for a long time (i.e., 10,000 cycles) under certain conditions, the authors indicated that eventually iron-based oxides will form, leading to higher wear [42]. In our study (i.e., for diamond against tungsten), tests were also performed with higher cycle numbers and did not show an increase in wear or any evidence of increased oxide formation. 

The lack of interactions between the diamond and tungsten surfaces has also previously been observed by the authors with molecular dynamic simulations [43]. Upon retraction of the surfaces in the MD simulations, no mechanical mixing (i.e., no bonding across the diamond/tungsten interface) was observed. This lack of bonding across the contact surfaces results in the low shear-stress response and correlates well with the microtribology experiments, which showed a reduction in the friction coefficient in UHV. In addition, subsequently to baking the chamber, the coefficient of friction approaches ultra-low values. This behavior is associated with the reduction of the water condensation on the surfaces, which minimizes the capillary forces and thus, the resistance to movement.

## 4. Conclusions

In this study, we investigated the interfacial phenomena of diamond—tungsten sliding contacts by means of an on-line tribometer followed by ex situ analysis. The macro-scale tribometry revealed a relatively low coefficient of friction, which correlates well with low roughness parallel to the sliding direction obtained throughout the sliding. Similarly, most of the wear occurred throughout the initial cycles of the test and approached low values during the steady state. Ex situ analysis was performed by means of XPS and SEM-FIB in order to better understand the underlying mechanisms of low friction and low-wear sliding. The analysis shows minimal amounts of tribofilm and transferfilm formation on both surfaces, which correlates well with the low friction values. In order to further elucidate the low friction of this tribocouple, unlubricated microscale sliding experiments of diamond on tungsten were performed under various environmental conditions. It was observed that the friction is significantly reduced in an ultrahigh vacuum compared to the 50% RH experiments, which was attributed to the reduction in the capillary forces. In addition, subsequently to baking the chamber, the coefficient of friction approached ultra-low values. Based on the results obtained in this study, the diamond/tungsten tribocouple seems promising for tribological interfaces in spacecraft systems, which can improve the durability of the components. 

## Data Availability

The data presented in this study are available on request from the corresponding author after obtaining permission of authorized person.
